# Mechanisms of chemoresistance in cancer stem cells

**DOI:** 10.1186/2001-1326-2-3

**Published:** 2013-01-17

**Authors:** Lissa Nurrul Abdullah, Edward Kai-Hua Chow

**Affiliations:** 1 Cancer Science Institute of Singapore, National University of Singapore, 14 Medical Drive #12-01, Singapore, 117599, Singapore; 2 Department of Pharmacology, Yong Loo Lin School of Medicine, National University of Singapore, Level 5, #05-09, 10 Medical Drive, Singapore, 117597, Singapore

**Keywords:** Chemoresistance, Cancer, Cancer stem cells

## Abstract

Chemotherapy is one of the standard methods of treatment in many cancers. While chemotherapy is often capable of inducing cell death in tumors and reducing the tumor bulk, many cancer patients experience recurrence and ultimately death because of treatment failure. In recent years, cancer stem cells (CSCs) have gained intense interest as key tumor-initiating cells that may also play an integral role in recurrence following chemotherapy. As such, a number of mechanisms of chemoresistance have been identified in CSCs. In this review, we describe a number of these mechanisms of chemoresistance including ABC transporter expression, aldehyde dehydrogenase (ALDH) activity, B-cell lymphoma-2 (BCL2) related chemoresistance, enhanced DNA damage response and activation of key signaling pathways. Furthermore, we evaluate studies that demonstrate potential methods for overcoming chemoresistance and treating chemoresistant cancers that are driven by CSCs. By understanding how tumor-initiating cells such as CSCs escape chemotherapy, more informed approaches to treating cancer will develop and may improve clinical outcomes for cancer patients.

## Review

Our understanding of cancer has changed over the years, owing to rapid advances in oncology research. The disease itself is not only characterized as a mass of excessive, uncontrolled growth of abnormal cells but is also defined by the dynamic alterations in the genome that cause cancer [1]. Left unchecked, cancer progression leads to disruption of normal biological processes via cellular invasion into local adjacent tissues and distal organs through metastasis. In addition to conventional cancer treatments such as surgery, radiation and cytotoxic chemotherapy, more selective treatments based on increased understanding of tumor biology and specific tumor subtypes have also become available [2]. Even with these advances in cancer therapy, chemotherapy remains an important component of cancer treatment. Currently, the complete elimination of cancer continues to elude oncologists as 90% of drug failures in metastatic cancers are attributed to chemoresistance [3]. Understanding the mechanisms by which chemoresistance can occur is important to developing novel therapeutic approaches to treating cancer.

In some cases, intrinsic chemoresistance may result in the survival of a population of tumor cells that subsequently leads to recurrence following treatment. This may be particularly true for tumors that are composed of a heterogeneous population of cells. For heterogenous tumors, the tumor initiating potential and drug sensitivity of different tumor cells within the same tumor bulk has yielded two models of cancer initiation: the stochastic model and the hierarchical model [4]. The stochastic model proposes that there is no variation in tumor initiating potential among different tumor subpopulations and that tumor cell growth is dependent on immune response, microenvironment and intrinsic gene regulatory signals. In contrast, the hierarchical concept suggests that different subpopulations of cells within a tumor have varying levels or absence of tumor initiating potential. Those fractions of cells that have enhanced tumor initiating potential are referred to as cancer stem cells (CSC). While CSC are not necessarily derived from normal stem cells, defining characteristics of CSCs include the ability to self-renew as well as differentiate into other tumor cell subtypes.

The hierarchical model of CSCs has been proposed for many decades and suggested as a mechanism for tumor initiation in both hematological malignancies as well as solid tumors such as breast cancer [5]. While it is now clear that not all heterogenous cancers follow the hierarchical model [6], there is growing evidence for a role of CSCs in a number of cancers. Early evidence for CSCs was first seen in hematological malignancies. In 1994, Lapidot et al. identified a subpopulation of tumor-initiating cells in acute myelogenous leukemia (AML) [7]. The identification of these leukemic initiating cells was based on differential expression of cell surface markers CD34 and CD38 where only CD34^+^/CD38^-^ AML cells could give rise to leukemic growth in severe combined immodeficiency (SCID) mice even though they represented a small fraction in the total leukemic population. Additionally, these cells demonstrated self-renewal and differentiation through recapitulating the entire hierarchy of human leukemia in a mouse. Thus, this work described important properties that are utilized now to identify CSCs, namely tumor-initiation, self-renewal and differentiation. This work led to one of the earliest descriptions of leukemic stem cells (LSCs) based on specific surface marker expressions and provided strong evidence for the existence of CSCs in cancer. While hematological malignancies provide the greatest evidence for the existence of CSCs, a number of studies have identified tumor-initiating CSCs in solid tumors as well. These CSCs were also identified based on phenotypic markers including surface protein expression and biological properties similar to those seen in normal stem and progenitor cells. This was demonstrated by Al-Hajj et al. when describing tumor-initiating breast CSCs [8]. In this study, primary human breast tumor cells were evaluated for tumor-initiating properties through orthotopic injection of these cells into mammary fat pads of NOD/SCID mice. This work identified ESA^+^CD44^+^CD24^-/low^ breast tumor cells as having greater tumorigenicity than cells lacking these markers. Furthermore, similar to studies with AML, secondary tumors from CSC xenografts contained heterogenous populations of tumor-initiating CSCs and non-tumorigenic daughter cells that lack these markers. Thus, these breast CSCs were capable of self-renewal and differentiation, hallmarks of both normal and cancer stem cells. Following this study, a number of reports have identified CSCs in solid tumors based on other surface markers such as CD133, EpCAM and CD90 as well as phenotypic markers such as side population (SP) or aldehyde dehydrogenase (ALDH) activity [9-13]. This includes the identification of solid tumor CSCs in a variety of organs including liver, brain, colon, pancreas, lung, ovaries and prostate [9-11,14-17].

The ability to identify and isolate CSCs in various tumor models has now led to the emergence of studies that are beginning to understand the mechanisms by which CSCs can contribute to tumor initiation as well as continued tumor progression. In some cases, CSCs appear capable of driving tumor population expansion and relapse following treatment through chemoresistance. While the mechanisms by which CSCs can escape chemotherapy treatment appear to be diverse, our studies suggest that these mechanisms may be influenced by specific oncogenes that are integral to a tumor’s initiation and subsequent growth [18]. In this review, we will discuss some of the mechanisms by which CSCs can escape chemotherapy as well as the clinical implications of these studies. Understanding the mechanisms by which CSCs can contribute to chemotherapy and tumor relapse is important as it provides important clues to better addressing cancer therapy and more specifically, cancer therapy that accounts for the unique biology of CSCs.

### ABC transporters and CSCs

Following chemotherapy, primary and metastatic sites of recurrence are often attributed to cells that have escaped chemotherapy. Because CSCs have been proposed to be the key tumor-initiating cell during recurrence, researchers have looked at chemoresistance as a functional mechanism by which one can identify and isolate CSCs. One such method that has proven useful in many different models of cancer is the identification of CSCs by enhanced efflux of Hoechst 33342 dye through ATP-binding cassette (ABC) transporters. Defined as side population (SP) cells during flow cytometry analysis, they were initially demonstrated to be useful in isolating hematopoetic stem cells [19]. One of the earliest studies demonstrating SP analysis as a method for enriching for CSCs occurred utilizing the C6 rat glioma cell line [12]. In this study, SP cells were demonstrated to be the key tumor-initiating CSCs for the C6 cell line. Additionally, SP cells could repopulate both SP and non-SP cells suggesting that these cells possess the hallmark properties of CSCs, namely self-renewal and differentiation. Following this study, SP analysis has been used to identify CSCs in a wide variety of solid tumors, including breast, colon, ovarian and hepatic cancers [20-23]. Recently, we identified that while SP is a useful method for enriching for CSCs in hepatic cancer, this mechanism of chemoresistance is not a universal feature of hepatic CSCs [18]. In fact, the presence of SP cells in tumors was found to be highly dependent on the driving genetic alterations of the tumor. In the case of our study, hepatic tumors driven by MYC, but not AKT and RAS, had a significant number of SP cells that appeared to enrich for chemoresistant tumor-initiating CSCs. This study provided evidence that while these mechanisms of chemoresistance appear common, they may differ depending on the driving genomic alterations of cancer. Understanding how such genomic alterations result in different cancer phenotypes amongst patients will allow clinicians to make more informed decisions when diagnosing and treating cancers unique to specific patients.

CSCs identified as SP cells exhibit chemoresistance related to the ABC transporter expressed in these cells. Two ABC transporters have been identified as capable of effluxing Hoechst 33342 dye and mediating the SP phenotype in CSCs as well as normal cells; P-glycoprotein (MDR1) and breast cancer resistance protein (ABCG2) [18,24,25]. ABC transporters are generally located in the cellular plasma membrane and function in normal biology to protect cells from harmful toxins and xenobiotics. MDR1 is primarily found in the kidney, adrenal glands, capillary blood vessels of the brain and also in the placenta [26]. In normal cells, MDR1 is usually present at low levels and is responsible in preventing the entry of foreign toxins into the growing fetus or sensitive organs such as the brain. ABCG2 is found in milk ducts of the mammary gland, hematopoietic stem cells and the blood brain barrier [27-29]. Some drugs, such as doxorubicin (Dox), are effluxed by both ABCG2 and ABCB1. SP cells exhibit chemoresistance to Dox regardless of which ABC transporter is expressed [30]. Other drugs, such as paclitaxel, can only be effluxed by MDR1 and not ABCG2 [18,30]. Thus, we have demonstrated that SP cells that express primarily MDR1 are more resistant to paclitaxel but not ABCG2-specific drugs such as SN-38 [18,31].

Depending on the ABC transporter that mediates the SP phenotype, one method of overcoming this mechanism of chemoresistance involves the use of specific inhibitors of ABC transporters. While clinical trials for general ABC transporters such as verapamil have been performed, these studies were ended due to the dose-limiting toxic side effects of these molecules [32]. More specific molecules related to individual ABC transporter pumps are currently being tested [33]. Another method for overcoming this chemoresistance that has also shown promise is the use of nanoparticle drug-delivery of chemotherapeutics. We previously demonstrated that conjugation of Dox to nanodiamonds impaired efflux of Dox in MDR1 overexpressing cells and can improve the efficacy of Dox therapy in Dox-resistant tumor models [34]. As such, while ABC transporters are a major mechanism of chemoresistance, there is evidence that specific and non-specific methods of overcoming ABC transporter pump activity may be useful for improving chemotherapy against CSCs.

### Aldehyde dehydrogenase related chemoresistance

In addition to the identification of CSCs by SP analysis, another reported functional marker of CSCs is ALDH activity. Enhanced ALDH activity also appears to confer resistance to specific chemotherapeutics as well. ALDH1 is a cytosolic enzyme that oxidizes aldehydes and converts them into carboxylic acids [35]. In addition to ALDH1, there are 16 other isoforms of ALDH in the human body that also localize to the mitochondria in addition to cytosol l [36]. While various isoforms are expressed throughout the body, the kidney and liver have been observed to express the highest levels of ALDH. In normal liver function, ALDH1 functions as a cytosolic retinal dehydrogenase that irreversibly converts retinol (vitamin A) into retinoic acid [37]. The importance of retinoic acids in embryonic development and stem/progenitor cell differentiation has led to the identification of high expression of ALDH in primitive hematopoietic progenitors as well as in embryonic multipotent neuronal stem cells [38-40].

Because ALDH activity has been linked to normal multipotent stem and progenitor cells, ALDH activity has been extensively analyzed in candidate CSCs as a potential marker for CSCs. Acute myeloid leukemic cells with elevated ALDH activity appeared to have better engraftment potential in NOD/SCID mice than their ALDH negative counterparts [13]. In a study of normal and malignant mammary cells, Ginestier et al. demonstrated that on average 8% of normal mammary epithelial cells had ALDH activity as measured by ALDEFLUOR-positive staining [41]. Furthermore, ALDEFLUOR-positive breast cancer cells that had ALDH activity were capable of forming xenograft tumors with as little as 500 cells. ALDEFLUOR-negative cells from the same tumor samples, however, were unable to form xenograft tumors with as many as 50,000 cells. When ALDEFLUOR-positive staining was combined with CD44^+^/CD24^-^ markers, as little as 20 breast cancer cells could form tumors. In addition to breast cancer, a number of other solid tumors such as lung, pancreas, prostate, liver and head and neck squamous cancer have also demonstrated some evidence of ALDH activity as a marker for CSCs [42-46].

Long before ALDH activity was used as a marker for identifying CSCs, the potential role of ALDH in chemoresistance had already been identified. In 1984, John Hilton identified a chemoresistant role for ALDH in a cyclophosphamide-resistant L1210 leukemic cell line [47]. Studying the mechanisms of cyclophosphamide-resistance, he identified that this cell line had unusually high levels of ALDH activity and that cyclophosphamide resistance could be reversed by inhibition of ALDH activity with disulfiram. Subsequent studies by Friedman et al. confirmed the role of ALDH-mediated cyclophosphamide resistance in medulloblastoma [48]. Since these initial studies, the ability of ALDH expression to confer resistance to cyclophosphamide has been demonstrated in other cancer systems and it is presumed that high ALDH activity is indicative of cyclophosphamide resistance in cancer and CSCs [49]. Thus, inhibition of ALDH activity can serve to sensitize CSCs to chemotherapeutics such as cyclophosphamide. A study of early passage colon cancer xenograft tumors revealed that treatment with cyclophosphamide resulted in the enrichment of ESA^+^CD44^+^ colon CSCs in the surviving tumor cells. Furthermore, these colon CSCs exhibited high levels of ALDH activity. Treatment of these ESA^+^CD44^+^ colon CSCs with ALDH inhibitors or *ALDH1A1*-targeted siRNA resulted in increased sensitivity to cyclophosphamide demonstrating that the chemoresistance seen in their model was specifically attributed to elevated ALDH activity [50]. In addition to conferring resistance to cyclophosphamide, ALDH1A1 knockdown experiments in human pancreatic adenocarcinoma suggest that ALDH may also be capable of mediating resistance to gemcitabine as well [51]. Continued studies with direct ALDH inhibitors or inhibitors of pathways that influence ALDH expression may provide useful tools in overcoming chemoresistance in CSCs or directly impairing CSC growth [44,52-54].

### Pro-survival BCL-2 protein family members in CSCs

Another mechanism of chemoresistance that has been well explored in CSCs is the role of B-cell lymphoma-2 (BCL-2) protein and its family members. The BCL-2 protein family has been identified as a group of proteins that play an integral role in maintaining the balance between cell survival and apoptosis. BCL-2 primarily mediates its pro-survival effects by binding to the pro-apoptotic proteins BCL2-associated-X-protein (BAX) and BCL-2 homologous antagonist killer (BAK) and impairing their ability to release apoptogenic proteins such as cytochrome c from the mitochondria [55]. Originally identified as a putative oncogene in acute B cell leukemia, BCL-2 is expressed in a number of different neoplastic cells as well as a variety of hematopoietic lineage cells [56,57]. In addition to BCL-2, there are four other pro-survival family members including B-cell lymphoma extra large (BCL-XL), BCL-2-like-2 (BCL-W), BCL-2-related protein A1A (BCL-A1A) and myeloid cell leukemia sequence-1 (MCL1) [58]. Knockout studies have revealed key roles for these proteins in normal biology that include the survival of a number of progenitor cells including renal epithelial progenitors, melanocyte progenitors, fetal erythroid progenitors, neuronal cells, sperm cells and hematopoietic stem cells [59-63]. In addition to their role in normal biology, pro-survival members of the BCL-2 protein family have been identified as critical primary or secondary oncogenic events during tumorigenesis. This was initially suggested during the identification of *BCL-2* by chromosomal translocation analysis that also revealed the abnormal chromosomal translocation of the *MYC* oncogene in an acute B-cell leukemia patient [56]. The role of BCL-2 in tumorigenesis was further confirmed in Eμ-*Myc*/Eμ-*Bcl-2* double transgenic mice where mice that overexpressed both MYC and BCL-2 became terminally ill from leukemia within 50 days while mice that solely expressed MYC required up to 100 days to succumb to malignancy [64].

Considering the potent effect of BCL-2 family members on tumorigenesis and cancer cell survival, their role in CSC biology has been extensively studied. Konopleva et al. demonstrated that quiescent leukemic CD34^+^ progenitor cells highly expressed BCL-2 and BCL-XL [65]. Furthermore, differentiation of these cells with all-trans retinoic acid resulted in lower expression of these pro-survival proteins as well as increased sensitivity to cytosine arabinoside [65]. Madjd et al. also showed that BCL-2 was highly expressed in CD44^+^/CD24^-/low^ breast CSCs [66]. While the mechanism of expression of these proteins is unclear in all cancer models and may result from chromosomal translocation, work in CSCs also suggests that these proteins can be expressed and affect chemoresistance through induction by other signaling pathways required for CSC survival. In CD133^+^ colon cancer stem cells, Todaro et al. demonstrated that interleukin-4 (IL-4) was produced and utilized in an autocrine manner. When CD133^+^ colon CSCs were treated with IL-4 neutralizing antibodies a decrease in BCL-XL as well as an increased sensitivity to oxaliplatin and 5-fluorouracil (5-FU) was observed [67]. Ma et al. demonstrated that the AKT/PKB signaling pathway regulated BCL-2 expression in CD133^+^ human HCC cancer cells [68]. In the human HCC cancer cell line Huh7, CD133^+^ CSCs appeared to express higher levels of BCL-2 than their CD133^-^ counterparts. Treatment of these Huh7 and PLC8024 HCC cell lines with Dox or 5-FU resulted in increased selection for chemoresistant CD133^+^ cells that expressed higher levels of both activated phosphorylated and BCL-2. Treatment with an AKT1 specific inhibitor resulted in the potent loss of BCL-2 expression in CD133^+^ cells as well as an increased sensitivity of these cells to Dox or 5-FU that was equivalent to their CD133^-^ counterparts suggesting that BCL-2 induction by AKT1 may be a mechanism by which CSCs can mediate chemoresistance. Another mechanism by which BCL-2 family members may be induced in CSCs is through Aurora-A, an oncogenic serine/threonine kinase that regulates cell cycle [69,70]. Analysis of CD133^+^CD29^+^CD20^-^ colorectal CSCs revealed that these cells expressed high levels of Aurora-A as well as BCL-2, MCL-1 and BCL-XL [69]. Knockdown of Aurora-A by shRNA resulted in a strong reduction of BCL-2 and MCL-1 expression and a moderate decrease in BCL-XL expression. Similar to work by Todaro et al. the decrease in pro-survival BCL-2 family member proteins was associated with increased sensitivity to oxaliplatin and 5-FU. This work offers yet another pathway by which CSCs may drive BCL-2-related chemoresistance and a potential therapeutic target for overcoming this chemoresistance.

### Role of CSC-related signaling pathways in chemoresistance

In addition to the roles that MYC and AKT1 may play in chemoresistance, there are a number of other signaling pathways that have been demonstrated to contribute to CSC biology, including chemoresistance. One such pathway is the WNT/β-catenin signaling pathway, which is required for normal stem and CSC self-renewal in a number of cell types [71-73]. In an early study of tumorigenic OV6+ HCC progenitor cells, chemical activation of the WNT pathway enhanced renewal of OV6+ hepatic CSCs whereas lentiviral microRNA knockdown of β-catenin impaired this self-renewal. These OV6+ hepatic CSCs also exhibited enhanced chemoresistance to cisplatin that could be reversed by lentiviral microRNA knockdown of β-catenin [74]. Similar studies demonstrate that WNT/β-catenin signaling pathway can also confer chemoresistance to 5-FU and Dox [75,76]. While the mechanisms by which the WNT pathway mediates chemoresistance in not completely clear in all these studies and likely varies amongst cell lines and tumor types, one potential mechanism is through the upregulation of ABC transporter pumps. In chemoresistant neuroblastoma cells, activation of the WNT pathway by FZD1 induced MDR1 and Dox resistance [76]. In c-kit^+^ ovarian CSCs, chemoresistance to cisplatin and paclitaxel was demonstrated to be mediated ABCG2. ABCG2 expression and chemoresistance to both cisplatin and paclitaxel could be reversed by β-catenin siRNA knockdown [77].

Another signaling pathway that appears to play a role in both CSC maintenance and chemoresistance is the Notch signaling pathway. The Notch signaling pathway has been identified to play an important role in a number of processes during tumor progression and metastases including tumor initiation, angiogenesis, epithelial-mesenchymal transition (EMT)-driven metastatic growth as well as self-renewal of cancer stem cells [78]. Recent evidence suggests that Notch may also contribute to certain mechanisms of chemoresistance in cancer stem cells. In multiple colon cancer cell lines, treatment with oxaliplatin induced Notch activation. Furthermore, siRNA knockdown of Notch 1 or γ-secretase inhibitor (GIS) treatment that prevents Notch pathway activation could sensitize colon cancer cells to oxaliplatin and prevent chemoresistance [79]. In addition to a potential role in colon cancer cells, Notch proteins have been identified to be upregulated in ovarian CSCs and GIS treatment appears to sensitize ovarian CSCs to cisplatin through inhibition of Notch maintenance of MDR1 expressing CSCs [80,81]. Notch also appears to contribute to chemoresistance in CD133+ glioma CSCs in coordination with another signaling pathway that has previously been identified to regulate self-renewal in both normal and cancer stem cells, the Sonic hedgehog (SHH) pathway [82,83]. Abnormal activation of the SHH pathway has been reported in a number of CSC models [84]. While the specific mechanisms are not clear, inhibition of the SHH pathway has been demonstrated to sensitize CSCs in a variety of tumor types including gastric cancer, pancreatic cancer, ovarian cancer and prostate cancer [84-87].

Regulators of inflammation, such as the NF-κB pathway, can also contribute to chemoresistance. A key mediator of the inflammatory response, NF-κB has a diverse set of biological function that can contribute to both pro-tumorigenic and anti-tumorigenic responses [88]. Constitutive activation of NF-κB and other pro-inflammatory signals in CD44^+^ ovarian CSCs appeared to correlate with chemoresistance to paclitaxel and carboplatin [89]. Inhibition of NF-κB by Eriocalyxin B induced cell death in chemoresistant CD44^+^ ovarian CSCs [90]. In breast CSCs, treatment with disulfiram and copper can inhibit consitutively active NF-κB while sensitizing these cells to paclitaxel [91]. There are likely a number of other signaling pathways that also can contribute to chemoresistance in CSCs that is likely dependent on the cell origin as well as other genetic alteration that drive the formation of these CSCs beyond those summarized in this review (Table 1).

**Table 1:** Summary of chemoresistance-related signaling pathways in this review

**Transcription factor/signaling pathway**	**Tumor type**	**Drug resistance**	**References**
MYC	Hepatocellular Carcinoma, Leukemia,	Paclitaxel, Doxorubicin,	18, 56, 64
AKT/PKB	Hepatocellular Carcinomaxx	Doxorubicin, 5-Fluorouracil	68
WNT/β-Catenin	Hepatocellular Carcinoma, Neuroblastoma, Ovarian Cancer	Cisplatin, Doxorubicin, 5-Fluorouracil, Paclitaxel	74, 75, 76, 77
Notch	Colon Cancer, Ovarian Cancer, Glioma	Oxaliplatin, Cisplatin, Temozolomide	79, 80, 81, 82
Sonic hedgehog	Glioma, Gastric Cancer, Pancreatic Cancer, Ovarian Cancer, Prostate Cancer	Temozolomide, Oxaliplatin, Gemcitabine, Paclitaxel, Cisplatin	82, 84, 85, 86, 87
NF-κB	Ovarian Cancer, Breast Cancer	Paclitaxel, Carboplatin,	88, 89, 90

### Altered DNA damage response in CSCs

A major mechanism that contributes to cancer progression and chemoresistance is an enhanced DNA damage response. Under hypoxic conditions, tumor cells can induce a potent DNA damage response primarily through hypoxia-inducible factor transcription factors [92]. Following this initial detection of hypoxia and response to DNA damage, two major signaling pathways are activated, ataxia telangiectasia mutated (ATM) and ATM and Rad-3-related (ATR) [93]. ATM and ATR can subsequently regulate cell cycle by phosphorylating downstream kinases checkpoint kinase 2 (CHK2) and checkpoint kinase 1 (CHK1), respectively. Following activation, ATM/CHK2 and ATR/CHK1 repressively phosphorylate cell division cycle 25 homolog B (CDC25B) and cell division cycle 25 homolog A (CDC25A). This in turn impairs CDC25 family member activation of cyclin dependent kinases (CDKs) and G_1_/S and G_2_/M transitions [94].

The very mechanisms that regulate cell cycle and promote DNA damage repair can also protect CSCs from DNA damaging radiation therapy and chemotherapeutics, particularly cytotoxic drugs that target tumor cell DNA. Analyzing CD133^+^ glioma stem cells, Bao et al. demonstrated that these cells were more resistant to ionizing radiation than CD133^-^ cells and could be enriched following radiation therapy [95]. Following radiation, CD133^+^ glioma stem cells exhibited much higher activated phosphorylation of DNA damage response factors ATM, CHK1 and CHK2 than CD133^-^ glioma cells. Furthermore, inhibition of CHK1/CHK2 with debromohymenialdisine reversed radioresistance in CD133^+^ glioma stem cells. Gallmeier et al. saw similar results in CD133^+^ colon CSCs where CD133^+^ colon CSCs appeared to be more resistant to DNA interstrands crosslinking (ICL) agents such as cisplatin than CD133^-^ colon cancer cells [96]. Treatment of colon cancer cells with ICL agents resulted in a more pronounced increase in phosphorylation of CHK1 in CD133^+^ colon CSCs compared with CD133^-^ colon cancer cells. A role for CHK1 in chemoresistance in these colon CSCs was demonstrated by inhibition of CHK1 by SB218078 resulting in increased sensitivity of CD133^+^ colon CSCs to cisplatin. Similar sensitization to gemcitabine with CHK1 inhibitors was also seen in chemoresistant CD24^+^CD44^+^ESA^+^ pancreatic cancer stem cells as well [97]. This work provided more evidence that inhibition of CHK1 and the DNA damage response may be an effective method for targeting and treating chemoresistant CSCs.

## Conclusion

**Figure 1:**
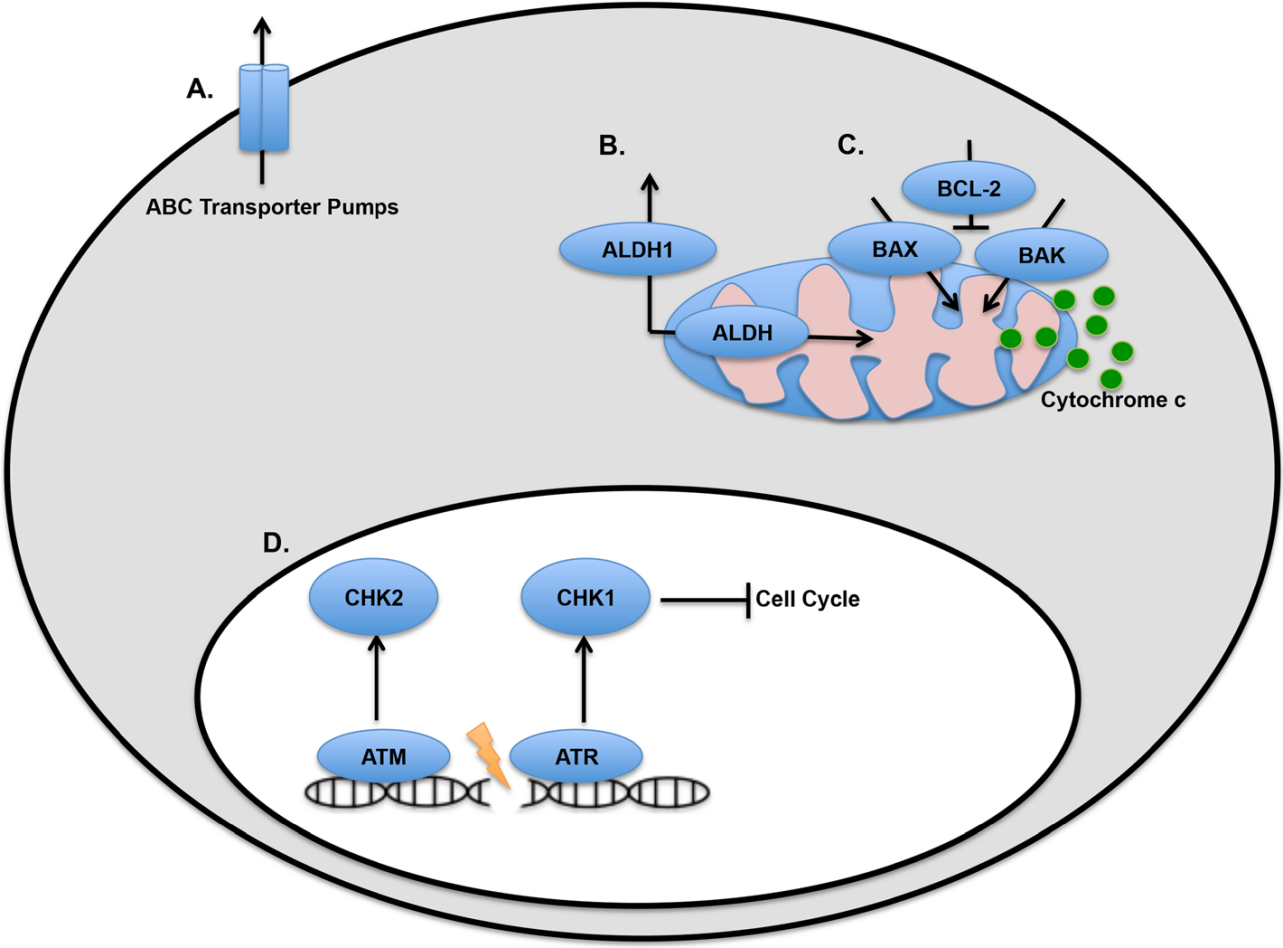
**Molecular Mechanisms of CSC Chemoresistance (A). ABC transporters can efflux a wide variety of chemotherapeutics out of cancer cells.** The specific ABC transporter pump expressed in the CSCs determines the specificity of chemoresistance. (**B**). ALDH1 is a cytosolic enzyme, whereas other isoforms can localize to the mitochondria as well as the cytosol. The efficacy of chemotherapeutic drugs such as cyclophosphamide is reduced in ALDH expressing CSCs, as these drugs are substrates for these enzymes. (**C**). Pro-survival protein BCL-2 binds to pro-apoptotic proteins BAX and BAK, preventing the release of the apoptogenic factor cytochrome c from the mitochondria. Aberrant activity of BCL-2 and other pro-survival BCL-2 family members utilize this mechanism to prevent chemotherapy-mediated apoptosis. (**D**). Following DNA damage, ATM and ATR recognize breaks in DNA and activate CHK2 and CHK1, respectively. CHK2 and CHK1 can impair cell cycle and promote DNA repair. Activation of these DNA repair proteins in CSCs can impair the efficacy of ICL agents.

CSCs can escape the toxic effects of chemotherapy through a variety of mechanism, including some not discussed in this review. Some of these mechanisms can be exploited as methods to diagnosis and identify CSCs while others have been previously identified as key mechanisms in overall tumor cell survival (Figure 1). Studies with specific oncogene models of cancer and studies of specific signaling pathways reveal that different signaling pathways and oncogenic factors can determine the mechanism by which CSCs mediate chemoresistance. Many of the studies highlighted in this review provide evidence that CSCs can be targeted and treated to improve overall therapy. As cancer treatment moves towards a more personalized medical approach, proper diagnosis paired with targeted and informed approaches to treating specific types of CSCs may prove to be a useful method for overcoming drug treatment failures that ultimately lead to recurrence and death.

## Abbreviations

CSC: Cancer stem cell; AML: Acute myelogenous leukemia; SCID: Severe combined immunodeficiency; LSCs: Leukemic stem cells; SP: Side population; ALDH: Aldehyde dehydrogenase; ABC: ATP-binding cassette; MDR1: P-glycoprotein; ABCG2: Breast cancer resistance protein; BCL-2: B-cell lymphoma-2; BAX: BCL2-associated-X-protein; BAK: BCL-2 homologous antagonist killer; BCL-XL: B-cell lymphoma extra large; BCL-W: BCL-2-like-2; A1A: BCL-2-related protein A1A; MCL1: Myeloid cell leukemia sequence-1; IL-4: Interleukin-4; 5-FU: 5-fluorouracil; Dox: Doxorubicin; GIS: γ-secretase inhibitor; SHH: Sonic hedgehog; ATM: Ataxia telangiectasia mutated; ATR: ATM and Rad-3-related; CHK2: Checkpoint kinase 2; CHK1: Checkpoint kinase 1; CDC25B: Cell division cycle 25 homolog B; CDC25A: Cell division cycle 25 homolog A; CDKs: Cyclin dependent kinases; ICL: Interstrands crosslinking.

## Competing interests

The authors declare that they have no competing interests.

## Authors’ contributions

EKC performed the oncogene-specific analysis of chemoresistant cancer stem cells that informed this review. EKC and LNA carried out the literature review necessary for this manuscript as well as the drafting of the manuscript. All authors reviewed and approved the final manuscript.
